# The Use of Granular Cyclopentanone as Alternative to Artificial Source of Carbon Dioxide in Improved Passive Outdoor Host Seeking Device (POHD)

**DOI:** 10.1155/2020/7620389

**Published:** 2020-06-13

**Authors:** Stella T. Kessy, Bruno A. Nyundo, Ladslaus L. Mnyone, Issa N. Lyimo

**Affiliations:** ^1^Department of Environmental Health and Ecological Sciences, Ifakara Health Institute, P.O. Box 53, Off Mlabani Passage, Ifakara, Morogoro, Tanzania; ^2^Zoology and Wildlife Conservation Department, College of Natural and Applied Science, University of Dar Es Salaam, P.O. Box 35091, Dar Es Salaam, Tanzania; ^3^Pest Management Centre, Sokoine University of Agriculture, P.O. Box 3110, Morogoro, Tanzania; ^4^School of Public Health, Faculty of Health Sciences, University of the Witwatersrand, Johannesburg, South Africa

## Abstract

Reliable sources of CO_2_ that are relatively cheap, obtainable, and easy to sustain are immediately required for scaling up of odor-baited mosquito surveillance and control devices. Several odor-baited devices are in the pipeline; however, their scale-up against residual malaria transmission, particularly in resource poor areas, is limited by the unavailability of reliable sources of CO_2_ and reliance on electrical power sources among other factors. We evaluated the use of granular cyclopentanone as an alternative to artificial or yeast fermentation-produced CO_2_ in passive outdoor host seeking device (POHD). Experiments were conducted against semifield reared *An. arabiensis* within the semifield system (SFS) at Ifakara Health Institute. Mosquitoes were tested against odor-baited POHDs augmented with yeast fermentation-produced CO_2_, granular cyclopentanone, attractive blends (Mbita or Ifakara), or their combinations. An insecticide, bendiocarb, was a killing agent used as a proxy for marking the mosquitoes visit the POHDs. Relative attractiveness of different treatment combinations was compared based on the proportion of dead mosquitoes that visited the POHD. The POHD augmented with granules of cyclopentanone alone was attractive to *An. arabiensis* as much as, or more than, POHDs augmented with yeast fermentation-produced CO_2_. The POHD baited with CO_2_ attracted more mosquitoes than those POHDs baited with synthetic blends alone; when these blends are combined with CO_2_, they attracted more mosquitoes than individual blends. More importantly, such POHDs baited with cyclopentanone attracted far greater proportion of mosquitoes than the POHD baited with either Mbita or Ifakara blend alone. The granular cyclopentanone strongly enhanced/potentiated the attractiveness of POHD baited with Mbita blends against mosquitoes compared to that of POHD baited with Ifakara blend. Moreover, the granular cyclopentanone retained its residual activity against *An. arabiensis* for up to 2 months after application particularly when used in combination with Mbita blend. In conclusion, this study demonstrates that cyclopentanone granules have the potential to substitute sources of CO_2_ in outdoor-based surveillance and control devices, thus warranting evaluation of such alternative under realistic field conditions.

## 1. Introduction

Mosquitoes play an overwhelming role in transmitting several vector-borne diseases to humans such as malaria, lymphatic filariasis, yellow fever, rift valley, dengue fever, Zika, and chikungunya [1–7]. Of these diseases, malaria causes the greatest health and socioeconomic burden [4, 6, 8], with 228 million cases and 405,000 deaths which are highly concentrated in sub-Saharan Africa [4, 6].

Mosquito vectors detect and locate their preferential blood meal sources mainly through chemoreception of volatile cues liberated from their hosts [9–14]. Mosquito antennae and maxillary palps have several receptors [10, 12, 15–18], which enable them to efficiently detect both the skin odors and carbon dioxide (CO_2_) plume from humans [13, 17, 19–24]. The CO_2_ is one of the most important long range attractants (normally detected at a range of 18–60 m) [21, 25, 26]; it attracts both the opportunistic (e.g., *An. arabiensis*) and anthropophilic mosquitoes (e.g., *Anopheles gambiae s.s.*, and *Culex quinquefasciatus*) [19, 27–29]. The combination of CO_2_ and skin odors has been shown to significantly attract high proportion of mosquitoes compared to CO_2_ or skin odor alone [11, 13, 14, 17, 20, 22, 30–32]. Therefore, CO_2_ is now widely used to enhance/potentiate the efficacy of odor-baited control and sampling devices [13, 30, 31, 33–40].

The existing odor-baited traps/devices rely on conventional sources of CO_2_ for sampling mosquitoes [41–43]. These conventional sources of CO2 include pressurized gas cylinders of industrial CO_2_ [30, 40], dry ice [33, 39, 44], and burning of propane [45]. Examples of traps baited with CO_2_ include BG-sentinel traps [40, 46], CDC light traps [39, 40], counterflow geometry traps [47], Mosquito Magnet-X traps (MMX-trap) [38], mosquito landing boxes [35], and mosquito trapping boxes [33]. However, these conventional sources of CO_2_ are expensive, unobtainable, and labor intensive to transport and sustain for large scale surveillance and control programs in resource poor areas [48]. Therefore, reliable sources of CO_2_ that are relatively less expensive, easy to use, and easy to sustain in remote poor resource settings are urgently required for use in odor-baited devices. Otherwise, the control of residual malaria transmission, which occurs almost exclusively outdoors, will remain a difficult endeavor.

Recently, several organic sources of CO_2_ have been proposed for use in mosquito traps for surveillance [32, 49, 50–52]. These organic sources of CO_2_ include yeast fermentation of sugar/sucrose and molasses [32, 52–54], electrolyzed oxalic acids (Harwood et al., 2014), granular CO_2_ sachets [55], and food-grade sources such as mixture of citric acid, water, and powdered sodium bicarbonate (NaHCO3), or vinegar (5%) mixed with sodium bicarbonate (NaHCO3) [56]. However, traps baited with CO_2_ from organic sources attract relatively fewer mosquitoes than those baited with industrial CO_2_ in pressurized gas cylinders or dry ice [55, 56]. These organic sources of CO_2_ are still expensive and logistically difficult to use for large scale surveillance and control programs because they require frequent replenishment of materials to extent residual activity. Therefore, the portable, easy to use, long-lasting, and cheaper novel sources of CO_2_ that may attract high proportions of mosquitoes, comparable to or higher than those attracted with traditional sources of CO_2_, are urgently required for large scale application of odor-baited devices in poor resource countries.

One of the desirable options could be the use of novel compounds that mimic CO_2_ such as cyclopentanone (C_5_H_8_O) [57, 58], acetone [59], and 2-butanone [60, 61]. These compounds activate CO_2_ receptor (cpA) neuron on the maxillary palps of mosquitoes, and some of them have been tested against *Anopheles*, *Culex*, and *Aedes* mosquitoes as substitutes for CO_2_ in odor-baited traps without success [17, 58, 60]. Traps baited with liquid formulation of cyclopentanone had similar catches of *Culex quinquefasciatus* to those traps baited with industrial CO_2_ under semifield conditions [17], but not in the field [58]. Similarly, the liquid formulation of 2-butanone increased attractiveness of synthetic blend (e.g., Mbita blend) to mosquitoes including *Anopheles gambiae* and *Anopheles funestus* the same as CO_2_ in the odor-baited traps under the field but not semifield environments [60]. However, it was hypothesized that liquid formulation of cyclopentanone or 2-butanone in a cotton wick may depreciate relatively quickly due to variations of wind speed, moisture, temperature, and competing odors from the surrounding natural vegetation [58, 60]. Therefore, there is a need for slow release formulations of cyclopentanone or other CO_2_ mimics such as 2-butanone to enhance further development and large scale use of traps and odor-baited outdoor control devices [17, 58, 60]. This study aimed to (i) evaluate the potential of cyclopentanone granules to enhance/potentiate the attractiveness of synthetic blends in POHD against *An. arabiensis*, (ii) compare the efficacy of POHD baited with granular and strip formulation of cyclopentanone against *An. arabiensis*, and (iii) assess the residual activity/persistence of such formulations against *An. arabiensis*.

## 2. Materials and Methods

### 2.1. Study Site

**Figure 1:**
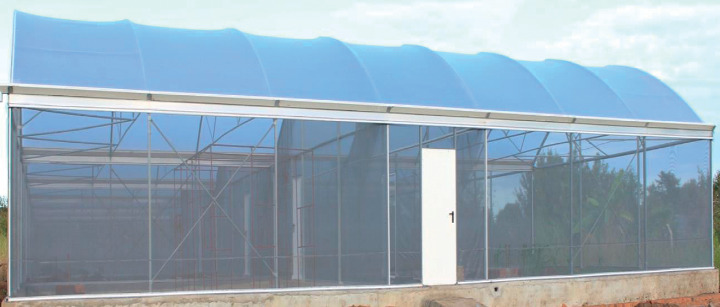
A picture of the semifield system (SFS). This SFS is located at Ifakara Health Institute in Kining'ina village, Kilombero Valley, Southeastern Tanzania.

Experiments were conducted within the semifield system (SFS) located at Kining'ina village in Kilombero Valley (Figure 1), about 6 km from Ifakara town. The SFS has several chambers (2.97 × 6.70 × 2.80 m) with temperature range from 26 to 32°C.

### 2.2. Mosquitoes

The colony of *An. arabiensis* was established in 2008 by collection of gravid females from the wild population at Sagamaganga village, Kilombero Valley (8.0667 S, 36.8000 E) [62]. This colony of mosquitoes is reared under ambient conditions in one chamber (3.30 × 2.70 × 2.50 m) of the semifield system (60 × 20 m) [62]. The temperature and relative humidity in this system range from 25 to 32°C and from 70 to 90%, respectively. The larvae are kept in rearing basins and are fed on TetraMin® (Tetra GmbH, Germany), as finely ground baby fish food flakes twice to three times a day. The adult mosquitoes are maintained inside the cages (0.45 × 0.45 × 0.45 m) with 10% glucose solution. Female *An. arabiensis* mosquitoes used throughout this study were 3–7 days old.

### 2.3. Improved POHD and Different Treatments

**Figure 2:**
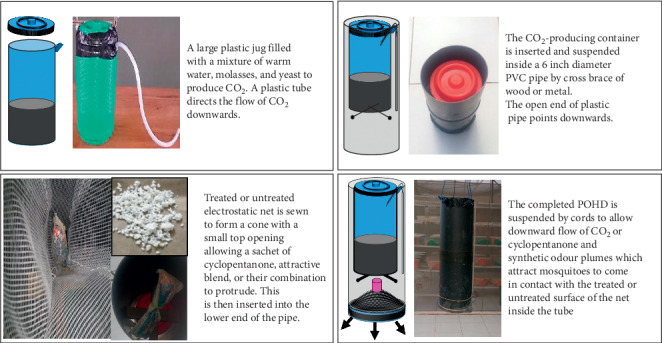
Schematic drawing and pictures showing improved passive host seeking device (POHD) with bottom placement of synthetic attractive blends and mosquito killing agent (bendiocarb). Components of the POHD are (a) the inner red jug containing mixture of warm water, molasses, and yeast for production of carbon dioxide (CO_2_) and a rubber tube for channelling CO_2_ downwind; (b) the CO2‐producing plastic jug is inserted and suspended inside 6-inch PVC pipe using fixed wood pieces; (c) a holed conical electrostatic netting untreated or treated with powdered bendiocarb and plugged with a bag/sachet containing granules of synthetic attractive blend, the granules of novel CO_2_ mimic compound, and 20% cyclopentanone as alternative to yeast fermentation-produced CO_2_; (d) a complete POHD with polyvinyl chloride (PVC) outer cover to allow downwind flow of odor and CO_2_ plumes to attract mosquitoes towards treated or untreated netting inside the device.

The improved POHD with bottom mosquito entrance was used in these experiments (Figure 2, Kessy et al., unpublished). Cyclopentanone, CO_2,_ and synthetic blend (Mbita or Ifakara) [36, 63] were deployed as attractants in the improved POHD, singly or in combination to compare their attractiveness against *An. arabiensis* female mosquitoes. The Mbita and Ifakara blends were designed and developed to either sachet containing granules or soaked nylon strips by Biogents AG, Germany. Two forms of CO_2_ were used: (i) the yeast fermentation-produced CO_2_ from modified mixture of yeast (8.75 g) and molasses (250 g) [50], and a total volume of 1 L of warm water based on previous studies that produced CO_2_ using at least 1 g of yeast in a total volume of ≤1 or >1 L of water [32, 52, 64, 65], (ii) 20% cyclopentanone (C_4_H_8_O) used in previous studies [17], which was modified and formulated to granules and impregnated nylon strips by Biogents AG, Germany. The synthetic attractive blends and cyclopentanone were stored in the refrigerator at 4°C between experiments. A powder formulation of bendiocarb (Ficam D) was applied on electrostatic charged netting [66] to kill mosquitoes visiting the improved POHD. Bendiocarb is a nonrepellent insecticide that acts by contact against mosquitoes including the population of *An. arabiensis* in Kilombero Valley [72]. These vector species are fully susceptible to bendiocarb (Lwetoijera et al., 2014; [72]; Matiya et al., 2019). Thus, bendiocarb was a suitable carbamate for use in POHD baited with lures to attract and kill any visiting mosquitoes and to quantify the number of dead mosquitoes as a proxy for attractiveness of the POHD to mosquitoes.

## 3. Experimental Procedures

### 3.1. Potentiating/Enhancing Effects of Cyclopentanone

**Figure 3:**
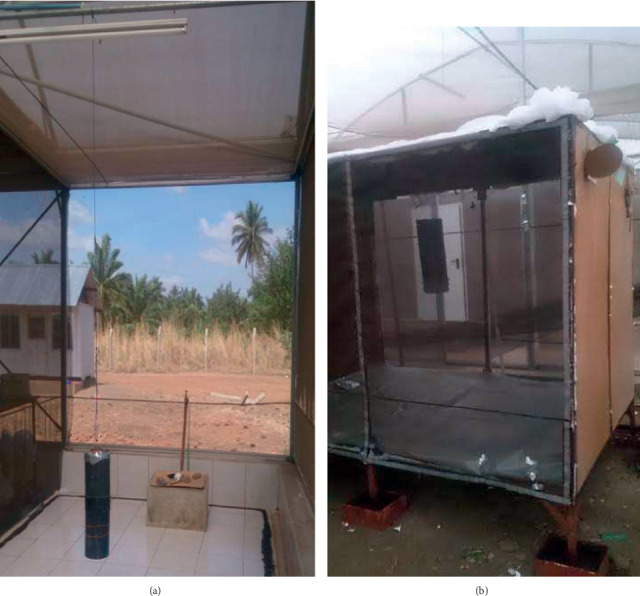
Improved passive host seeking device (POHD) was assembled and baited with attractants, combined with or without the mosquito killing agent (bendiocarb). This device was exposed to *An. arabiensis* by hanging in the middle of (a) chamber of the SFS and (b) rectangular bioassay box.

The sachets of granular formulation or impregnated nylon strips of either Mbita (Mb) or Ifakara blend (Ib) were hung inside the improved POHD. Eight (8) different treatment combinations were evaluated: (1) CO_2_ + cyclopentanone + odor blend + untreated netting (untreated), (2) bendiocarb-treated netting alone (Be), (3) odor blends + Be (Mb or Ib), (4) CO_2_ + Be (CO), (5) cyclopentanone + Be (Cy), (6) CO_2_ + odor blends + Be (MbCO or IbCO), (7) cyclopentanone (Cy) + odor blends + Be (CyMb, or CyIb), and (8) odor blend + CO_2_ + Cy + Be (CyMbCO or CyIbCO). The improved POHD components were assembled and hung at the middle of the chamber within the SFS, 0.25 m off the ground (Figures 2 and 3(a)). In each experiment, a total of 100 starved female mosquitoes (25 individuals per cup) were released at four different corners of the SFS against the aforesaid treatment combinations. Mosquitoes were left to forage for overnight. The next morning, all live or dead mosquitoes from inside the POHD and other parts of the SFS chamber were independently recovered, counted, and recorded. All live mosquitoes were kept in the semifield insectary, provided with 10% glucose solution, and monitored for mortality after 24 hrs. Treatment combinations were randomly alternated between the days of the experiments. Between experiments, any mosquitoes remaining inside the experimental SFS chamber were removed using CDC backpack aspirator to avoid spillover effect. These experiments were replicated three times for each treatment combination.

### 3.2. Persistence of Granular Cyclopentanone

Persistence of cyclopentanone was evaluated using a rectangular bioassay box (1.87 × 2.12 × 1.15 m), inside three different SFS chambers (2.50 × 9.50 × 9.00 m), one box per chamber (Figure 3(b)). The synthetic blend that was strongly enhanced by granular cyclopentanone from the efficacy experiment above, Mbita blend in this case, was selected to be combined with cyclopentanone in these experiments. Persistence was counted from the day the cyclopentanone started being used after preparation, and month is abbreviated as “mo” in the text and the figures. The treatment combinations were as follows: (1) fresh granules of cyclopentanone (Cy) + Mbita blend (Mb) + untreated netting (untreated), (2) fresh granules of Mbita blend alone (Mb), (3) 2 mo old granules of cyclopentanone + bendiocarb-treated netting-Be (2moCy), (4) 2 mo old granules of cyclopentanone + Mb + Be (2moCyMb), (4) fresh granules of cyclopentanone + Be (FreshCy), and (5) fresh granules of cyclopentanone + Mb + Be (FreshCyMb). Then, improved POHD was assembled and hung at the middle of the rectangular box at 0.25 m off the ground (Figure 3(b)). The two sides of the box were closed with cardboard, but two holes (0.11 cm in diameter) were made for the release of mosquitoes inside during experiments. Each time of the experiment, a total of 100 female mosquitoes starved for 6 hours without glucose were released inside the box through 4 holes (25 individual mosquitoes/holes × 4 holes = 100 mosquitoes) in the evening at 7:00 pm. Recapture and monitoring of mosquitoes were done following the same procedures as in the efficacy experiments above. These experiments were replicated three times for each treatment combination.

### 3.3. Comparing Efficacy of Different Formulations of Cyclopentanone

These experiments were conducted using rectangular bioassay boxes (in experiment 2 above), with the aim of comparing the efficacy of cyclopentanone applied on granules and nylon strips delivery formats against mosquitoes. The formulations of cyclopentanone were tested in combination with long-lasting granular formulation of Mbita blend (depicted from persistence experiments). Different treatment combinations evaluated in the improved POHD were as follows: (1) fresh cyclopentanone + Mbita blend + untreated netting, (2) fresh cyclopentanone strip + Mb + Be (strips), and (3) fresh cyclopentanone granules + Mb + Be (granules). The improved POHDs incorporated with different treatment combinations were assembled (independently) and hung at the center of the box (Figure 3(b)). Mosquitoes were exposed to improved POHDs, recaptured, and monitored for 24-hour mortality, in the same way described in experiments 1 and 2 above. These experiments were replicated three times for each treatment combination.

### 3.4. Ethical Considerations

The research team and technical assistants responsible for rearing and handling mosquitoes were routinely (i.e., after every 7 days) screened for malaria to ensure that experimental materials are free of malaria parasites. The semifield had double doors, and it was routinely checked to ensure that the screens were always intact to prevent escape of mosquitoes to the environment. The remains of bendiocarb and/or treated materials were disposed accordingly and thereafter incinerated. The ethical review and approval were granted by the Institutional Ethics Review Board (IRB) of Ifakara Health Institute (Ref: IHI/IRB/No: 14-2013) and the Medical Research Coordinating Committee at the National Institute of Medical Research in Tanzania (NIMR/HQ/R.8a/Vol. IX/1784). This work was also granted permission for publication by National Institute of Medical Research in Tanzania (NIMR/HQ/P.12/Vol. XXVIII/93).

### 3.5. Statistical Analysis

Statistical analyses were conducted to test the efficacy of cyclopentanone as alternative to CO_2_ for enhancing/potentiating the attractiveness of synthetic blends, to determine the duration of their effects (persistence), and to compare between nylon strips and granular formulations against *Anopheles arabiensis*. Response variable measured in these experiments was proportion of mosquitoes killed by different treatments of POHD. The response variable of proportion of dead mosquitoes was analyzed using generalized linear mixed effect models with binomial errors (glmer) in the R statistical software package [71]. The explanatory variables, “treatments,” “blend type,” and “formulations,” were considered as the main effects, whereas “days” of the experiments were considered as random effects. A base model including only random effect of “day” was constructed. A sequential addition of the “main effects” and their interaction (treatment × blends or treatment formulation) to the base model was conducted to construct a maximal model (forward stepwise approach). A statistical significance of fixed effects and interaction term was generated and evaluated using likelihood ratio tests (LRTs). When the interaction terms were statistically significant, the main effect of either “blend type” or “formulation type” for each synthetic attractant was analyzed separately to generate estimates for the main effects. Then, the full model was used to perform a two-ways multiple comparisons using Tukey post hoc tests (adjusting for multiple comparison) to establish statistical significant differences between treatments.

## 4. Results

### 4.1. Potentiating/Enhancing Effect of Cyclopentanone

**Figure 4:**
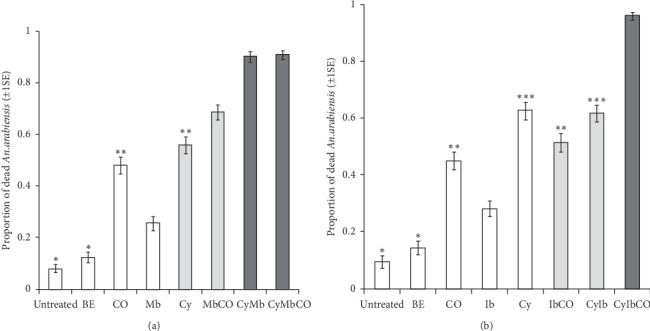
Estimated proportion (±1 s.e.) of *An. arabiensis* mosquitoes that were killed after exposure to untreated or bendiocarb-treated improved passive host seeking device baited with attractants within the semifield system: (a) Mbita blend (Mb) and cyclopentanone (Cy), (b) Ifakara blend (Ib) and cyclopentanone (Cy). The statistical significant differences between treatments are indicated as follows: the black boxes indicate that the two treatments are similar but statistically significantly different from all other treatments; the grey boxes indicate that the two are similar but statistically significantly different from all others; the different numbers of asterisks indicate statistical significant differences between treatments; the open boxes without asterisks indicate statistical significant difference from all other treatments. The treatments are abbreviated as follows: untreated: attractants without bendiocarb-treated netting, BE: bendiocarb-treated netting without attractants, CO: carbon dioxide (CO_2_), Mb: Mbita blend, Cy: cyclopentanone granules, MbCO: Mbita blend combined with CO_2_, CyMb: Mbita blend combined with cyclopentanone granules, CyMbCO: Mbita blend combined with cyclopentanone granules and CO_2_, IbCO: Ifakara blend combined with CO_2_, CyIb: Ifakara blend combined with cyclopentanone granules, CyIbCO: Ifakara blend combined with cyclopentanone granules and CO_2_. Error bars represents plus/minus 1 standard error.

The attractiveness of improved POHD against *Anopheles arabiensis* was influenced by interaction between treatments and attractive blend types (treatment ∗ blend type: *χ*_2_^2^ = 2.56, *P* < 0.001, Figures 4(a) and 4(b), Tables 1 and 2). Overall, all treatments combinations of POHD baited with Mbita blend (Mb) including augmentation with cyclopentanone (Cy) and carbon dioxide (CO) attracted and killed more mosquitoes than those of POHD baited with Ifakara blend (Ib). However, the two blends (Mbita and Ifakara blend), when acting alone without augmentation, attracted similar proportions of *An. arabiensis* to improved POHD (*χ*_1_^2^ = 2.56, *P* < 0.001, Figures 4(a) and 4(b), Table 1).

The attractiveness of improved POHD baited with Mbita blend to *An. arabiensis* varied significantly between treatments (*χ*_7_^2^ = 44.629, *P* < 0.001, Figure 4(a), Tables 1 and 2). The improved POHD baited with attractants alone (i.e., without bendiocarb) or bendiocarb-treated nettings without attractants acted as controls which killed similar proportion of *An. arabiensis* (*z* = 1.68, *P*=0.69, Figure 4(a), Table 1). The bendiocarb-treated POHD without attractants killed significantly fewer mosquitoes than bendiocarb-treated POHD baited with CO (*z* = 8.5, *P* < 0.001), Mb (*z* = 3.69, *P* < 0.001), Cy (*z* = 9.47, *P* < 0.001), MbCO (*z* = 11.64, *P* < 0.001), CyMb (*z* = 14.34, *P* < 0.001), and CyMbCO (*z* = 14.98, *P* < 0.001) (Figure 4(a), Table 1). Similarly, the POHD baited with all three attractants but without bendiocarb-treated nettings attracted and killed fewer mosquitoes than the POHD baited with CO (*z* = −9.13, *P* < 0.001), Mb (*z* = −5.15, *P* < 0.001), Cy (*z* = −10.29, *P* < 0.001), MbCO (*z* = −12.23, *P* < 0.001), CyMb (*z* = −14.81, *P* < 0.001), and CyMbCO (*z* = −15.38, *P* < 0.001) (Figure 4(a), Table 1). When CO_2_ was applied alone in bendiocarb-treated POHD, it attracted and killed greater proportion of mosquitoes than POHD baited with Mb alone (*z* = 5.13, *P* < 0.0010), but it killed fewer mosquitoes than POHD baited with MbCO (*z* = 4.61, *P* < 0.001) (Figure 4(a), Table 1). Furthermore, the CO_2_ enhanced attractiveness of POHD baited with Mbita blend to mosquitoes; the POHD baited with MbCO attracted and killed greater proportions of mosquitoes than POHD baited with Mb alone (*z* = 9.32, *P* < 0.001) (Figure 4(a), Tables 1 and 2). In comparison with cyclopentanone, the bendiocarb-treated POHD baited with CO_2_ alone attracted and killed mosquitoes the same as POHD baited with Cy alone (*z* = 1.73, *P*=0.65), but it killed fewer mosquitoes than POHD baited with CyMb (*z* = 9.07, *P* < 0.001) and CyMbCO (*z* = 9.69, *P* < 0.001) (Figure 4(a), Tables 1 and 2). In fact, cyclopentanone enhanced attractiveness of POHD to mosquitoes. The bendiocarb-treated POHD baited with Cy alone attracted and killed similar proportions of mosquitoes to POHD baited with MbCO (*z* = 2.89, *P*=0.07, Figure 4(a), Tables 1 and 2), but it killed greater proportion of mosquitoes than POHD baited with Mb alone (*z* = 6.71, *P* < 0.001, Figure 4(a), Tables 1 and 2). Furthermore, the bendiocarb-treated POHD baited with Mbita blend killed more mosquitoes when combined with cyclopentanone. The bendiocarb-treated POHD baited with CyMb attracted and killed similar proportions of mosquitoes to POHD baited with CyMbCO (*z* = 0.25, *P*=1, Figure 4(a), Table 1), but it killed greater proportion of mosquitoes than POHD baited with Mb alone (*z* = −12.52, *P* < 0.001, Figure 4(a), Tables 1 and 2) and MbCO (*z* = −5.59, *P* < 0.001, Figure 4(a), Tables 1 and 2). Similarly, the bendiocarb-treated POHD baited with CyMbCO killed more mosquitoes than POHD baited with Mb alone (*z* = −13.22, *P* < 0.001, Figure 4(a), Table 1) and MbCO (*z* = −6.08, *P* < 0.001, Figure 4(a), Table 1).

Similarly, the attractiveness of POHD baited with Ifakara blend to *An*. *arabiensis* varied significantly between treatments (*χ*_7_^2^ = 48.79, *P* < 0.001, Figure 4(b)). The bendiocarb-untreated POHD baited with attractants and the bendiocarb-treated POHD without attractants were the controls for the combination of treatments which attracted and killed similar proportion of mosquitoes (*z* = 1.34, *P* = 0.87, Figure 4(b), Table 1). The bendiocarb-treated POHD without attractants (BE) killed relatively lower proportion of *An. arabiensis* than bendiocarb-treated POHD baited with different treatment combinations including CO (*z* = 6.89, *P* < 0.001), Cy (*z* = 9.79, *P* < 0.001, Figure 4(b)), Ib (*z* = 3.51, *P* < 0.001, Figure 4(b)), IbCO (*z* = 7.09, *P* < 0.001, Figure 4(b)), CyIb (*z* = 9.72, *P* < 0.001, Figure 4(b)), and CyIbCO (*z* = 13.25, *P* < 0.001, Figure 4(b)). Likewise, the bendiocarb-untreated POHD baited with all three attractants killed fewer proportions of mosquitoes than bendiocarb-treated POHD baited with CO (*z* = −6.93, *P* < 0.001), Cy (*z* = −9.30, *P* < 0.001), Ib (*z* = −4.27, *P* < 0.001), IbCO (*z* = −7.71, *P* < 0.001), CyIb (*z* = −9.21, *P* < 0.001), and CyIbCO (*z* = −13.01, *P* < 0.001). The yeast fermentation-produced CO_2_ applied to bendiocarb-treated POHD enhanced attractiveness of this device to *An. arabiensis*; consequently, this POHD killed similar proportion of these mosquitoes as bendiocarb-treated POHD baited with IbCO (*z* = 1.41, *P* = 0.84), but it killed more mosquitoes than POHD baited with Ib alone (*z* = −3.91, *P* = 0.002, Figure 4(b), Tables 1 and 2). In addition, the bendiocarb-treated POHD baited with IbCO attracted and killed more mosquitoes than POHD baited with Ib alone (*z* = 5.15, *P* < 0.001, Figure 4(b), Tables 1 and 2). In comparison with cyclopentanone, the POHD baited with CO_2_ alone attracted and killed significantly lower proportion of mosquitoes than POHD baited with different treatment combinations of cyclopentanone such as Cy alone (*z* = 3.96, *P* = 0.001, Figure 4(b), Tables 1 and 2), CyIb (*z* = 3.79, *P* = 0.003, Figure 4(b), Tables 1 and 2), and CyIbCO (*z* = −9.83, *P* < 0.001, Figure 4(b), Table 1). Indeed, cyclopentanone enhanced attractiveness of POHD to *An. arabiensis* when applied alone or in combination with Ifakara blend. The bendiocarb-treated POHD baited with Cy alone lured and killed similar proportion of these mosquitoes to POHD baited with CyIb (*z* = −0.23, *P* = 1) and IbCO (*z* = −2.52, *P* = 0.17), but it killed more mosquitoes than POHD baited with Ib alone (*z* = −7.47, *P* < 0.001). The bendiocarb-treated POHD baited with CyIb attracted and killed similar proportion of mosquitoes to POHD baited with IbCO (*z* = −2.34, *P* = 0.26, Figure 4(b)), but its attractiveness to mosquitoes was greater than that of POHD baited with Ib alone (*z* = −7.37, *P* < 0.001, Figure 4(b), Tables 1 and 2). Furthermore, bendiocarb-treated POHD baited with all three attractants (CyIbCO) strongly attracted and killed more mosquitoes than POHD baited with Ib alone (*z* = −11.74, *P* < 0.001, Figure 4(b), Table 1), IbCO (*z* = −9.04, *P* < 0.001, Figure 4(b), Table 1), and CyIb (*z* = 7.81, *P* < 0.001).

### 4.2. Persistence of Granular Formulation of Cyclopentanone

**Figure 5:**
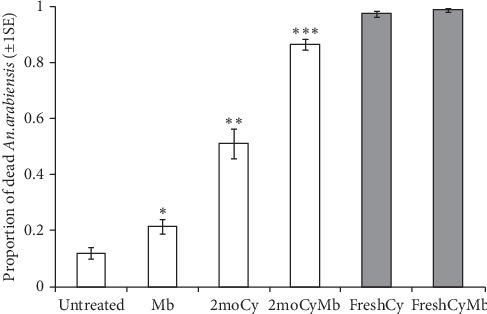
Estimated proportion (±1 s.e.) of *An. arabiensis* mosquitoes that were attracted and killed after exposure to an improved passive host seeking device that was untreated or treated with bendiocarb (BE) and baited with fresh or 2-month-old granules of cyclopentanone (Cy) or their combinations with fresh granules of Mbita blend (Mb) in a rectangular bioassay box within the semifield system. The statistical significant differences between treatments are indicated as follows: the black boxes indicate statistical significant differences from all other treatments; the different numbers of asterisks indicate statistical significant differences between treatments; no asterisk or color indicates statistical significant differences from all other treatments. The treatments are abbreviated as follows: untreated: attractants without bendiocarb-treated netting, Mb: Mbita blend, 2moCy: 2-month-old granules of cyclopentanone, 2moCyMb: 2-month-old granules of cyclopentanone combined with Mbita blend, FreshCy: fresh granules of cyclopentanone, and fresh CyMb: fresh granules of cyclopentanone combined with Mbita blend. Error bars represents ±1 standard error.

The attractiveness of POHD against *Anopheles arabiensis* was influenced by the persistence of cyclopentanone granules (*χ*_4_^2^ = 34.48, *P* < 0.001, Figure 5). Bendiocarb-treated POHD baited with fresh granules of cyclopentanone (FreshCy) or their combination with granules of Mbita blend (FreshCyMb) attracted and killed similar proportions of mosquitoes (*z* = 1.26, *P*=0.79, Figure 5). However, bendiocarb-treated POHD baited with 2-month-old granular cyclopentanone (2moCy), or 2-month-old granular cyclopentanone combined with Mbita blend (2moCyMb) attracted and killed significantly lower proportion of mosquitoes than that of POHD baited with fresh granular cyclopentanone (FreshCy) and their combination with Mbita blend (*P* < 0.001, in all cases, Figure 5). The POHDs baited with 2moCy, 2moCyMb, FreshCy, and FreshCyMb attracted and killed significantly greater proportion of mosquitoes than POHD baited with Mbita blend alone (*P* < 0.001, in all cases, Figure 5). However, POHD baited with 2moCy attracted and killed significantly fewer mosquitoes than POHD baited with combination of 2moCy and Mbita blend (*z* = 8.51, *P* < 0.001, Figure 5). Moreover, POHD without bendiocarb attracted and killed significantly fewer mosquitoes than all other POHDs baited with cyclopentanone alone or cyclopentanone combined with Mbita blend regardless of the age of the granules (*P* < 0.001, in all cases, Figure 5).

### 4.3. Efficacy of Different Formulations of Cyclopentanone

**Figure 6:**
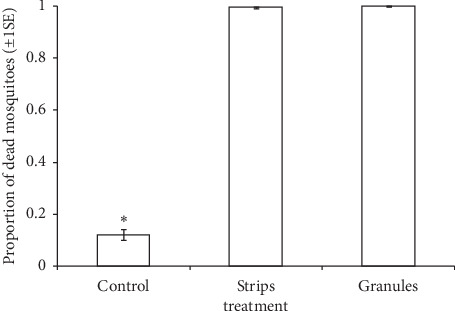
Estimated proportion (±1 s.e.) of *An. arabiensis* mosquitoes that were attracted and killed after exposure to an improved passive host seeking device that was untreated or treated with bendiocarb (BE) and baited with impregnated nylon strips or granular formulations of cyclopentanone (Cy) in combination with Mbita blend (Mb) in a rectangular bioassay box within the semifield system. The treatments were abbreviated as follows: control: attractants without benthiocarb-treated nettings, strips: cyclopentanone impregnated nylon strips, granules: granular cyclopentanone. The statistical significant differences between treatments are indicated as follows: the asterisks indicate statistical significant differences from all other treatments with open boxes without asterisks. Error bars represent ±1 standard error.

The efficacy of POHD baited with cyclopentanone varied significantly between treatments (*χ*_2_^2^ = 16.16, *P* < 0.001, Figure 6). The POHD without bendiocarb attracted and killed significantly lower proportion of mosquitoes than bendiocarb-treated POHD baited with granular cyclopentanone (*z* = 10.59, *P* < 0.001, Figure 6) and cyclopentanone impregnated nylon strips (*z* = 14.60, *P* < 0001, Figure 6). However, POHD baited with cyclopentanone impregnated nylon strips attracted and killed similar proportion of mosquitoes to POHD baited with cyclopentanone granules (*z* = −2.27, *P*=0.05, Figure 6).

## 5. Discussion

This study revealed the potential of granular formulation of cyclopentanone as an alternative to artificial CO_2_ applied in odor-baited traps against *An. arabiensis* that maintains residual transmission of malaria in most of African countries. The improved POHD baited with granular cyclopentanone was attractive as much as, or more than, the POHD baited with fermented CO_2_ to *An. arabiensis*. Plumes of both the granular cyclopentanone and CO_2_ act in the same way on mosquitoes CO_2_ receptors (cpA). This finding compares with the results of recent semifield and field experiments that tested liquid formulations of 2-butanone combined with nylon strips of Mbita blend [60] and previous semifield experiment that used liquid formulation of 20% cyclopentanone [17]. However, these results were both different from that of the field trial conducted by Philippe-Janon et al. [58]. Such contrast was attributed to the quick loss of attractiveness by liquid formulation cyclopentanone under field settings consequent to strong wind as well as fluctuations on temperature and relative humidity. Therefore, the slow release formulation (granules) of cyclopentanone offers an exciting option for use in odor-baited mosquito surveillance and/or control devices. Sachet of granular cyclopentanone is less bulky and easy to apply and distribute in large scale surveillance compared to other sources of CO_2_ such as pressurized cylinder of CO_2_ and gallons for yeast fermentation-produced CO_2_, and it lasts relatively longer than liquid formulations [17, 58, 60]. Thus, it can be deployed in resource poor areas like rural Tanzania. Although production of CO_2_ from a mixture of yeast and molasses was in certain circumstances as effective as, or less effective than, cyclopentanone, it has a drawback in that the mixture of materials in portable containers can release CO_2_ for approximately 8–12 hours and resources are highly needed for domestic consumption and logistically difficult to change and distribute on daily basis for large scale surveillance and control of mosquitoes.

Also, this study indicated that granular cyclopentanone had stronger potentiating/enhancing effect on synthetic blends than that of the yeast fermentation-produced CO_2_. Such effect of cyclopentanone on synthetic blends varied between blend types applied on the POHD. Nevertheless, the granular formulation of cyclopentanone enhanced/potentiated attractiveness of POHD baited with Mbita blend to *An. arabiensis* by 70%, but it enhanced the attractiveness of POHD baited with Ifakara blend by 56%. Previous semifield and field experiments indicated that Mbita blend is relatively more attractive to mosquitoes than Ifakara blend when augmented with CO_2_ [63]. The current study also found that the combinations of either blend with cyclopentanone or CO_2_ in POHD attracted far greater proportion of mosquitoes relative to the individual blends, Cy alone, or CO_2_ alone. This observation that CO_2_ enhances attractiveness of odor blends is also emphasized in several studies conducted elsewhere [11, 16, 30, 37, 38, 43]. The results of this study also confirms the recent findings that traps baited with Mbita blend and augmented with CO_2_ mimic compounds such as 2-butanone attracted similar number of mosquitoes as those traps baited with odor blends combined with CO_2_ gas [60]. Since plumes of cyclopentanone and CO_2_ act in the same way on mosquito's CO_2_ receptors [17], they may be used to augment synthetic blends in POHD and other devices of the sort.

Moreover, the residual activity of 2-month-old granular cyclopentanone was reduced by 47% relative to the fresh formulation when applied in POHD. Nevertheless, when such formulation was complemented with Mbita blend, the efficacy was elevated to 76%. Such stable residual activity of cyclopentanone could have been contributed by their storage under refrigeration temperature between experiments and slow release granular formulation. This slow release formulation may have enabled the older cyclopentanone to retain its enhancement/potentiation effects on synthetic blends in the POHD that attracted and killed >80% of the exposed mosquitoes. Similarly, previous studies showed that the application of BG lures as granules increased their residual activity for up to 5 months [34, 72]. These findings therefore indicate that attractiveness of cyclopentanone granules lasts for relatively longer time after application than other CO_2_ sources and thus will need to be replaced less frequently when applied in odor-baited traps for large scale vector surveillance and control.

On the other hand, fresh granules and nylon strips of cyclopentanone were equally effective in enhancing attractiveness of POHD baited with Mbita blend against *An. arabiensis*. The possible explanation could be that these experiments were conducted within a small sized chamber (1.87 × 2.12 × 1.15 m) where cyclopentanone with high volatility, when used alone or combined with Mbita blend, yielded equal concentration of odor plumes in a short range/distance to mosquitoes regardless of the formulations. Therefore, mosquitoes equally detected the plumes from different treatments. Variation between the two formulations could have been observed if they were compared under natural field conditions. Previous field work demonstrated that traps baited with nylon strip impregnated with Mbita blend combined with CO_2_ attracted as much *Aedes* and *Culex* mosquitoes as the traps baited with combination of long-lasting granular formulation of BG lures and CO_2_ [37]. Contrastingly, the recent study demonstrated that fresh liquid formulation of 2-butanone combined with synthetic odor blend attracted more *Anopheles* mosquitoes to the traps than traps baited with odor blend alone in the field, but not under the semifield conditions [60].

Although impregnated nylon strips of synthetic blends have been shown to remain attractive to mosquitoes for several weeks to months [34, 35, 43, 63, 73], they are not suitable for mass production and use in large scale mosquito surveillance and control programs [54]. Overall, the granular formulation of cyclopentanone may be portable, durable, lightweight, and easy to produce and use in odor-baited devices for large scale surveillance and control of mosquitoes. However, further investigations are required to assess long-lasting enhancing effects of granular formulations of cyclopentanone on synthetic blends in natural environments.

In conclusion, the present study demonstrates that cyclopentanone has the potential to substitute artificial source of CO_2_ in odor-baited devices for surveillance and control of *An. arabiensis*, thus warranting evaluation of such alternative under realistic field conditions.

## Figures and Tables

**Table 1:** Summary of numbers of *An. arabiensis* mosquitoes exposed and their responses to the improved POHD baited with attractants alone or their different treatment combinations.

Types of blend	Combination of treatments	Total number of exposed mosquitoes	Total number of dead mosquitoes	Mean number of dead mosquitoes
Mbita blend (Mb)	CyMbCO	274	249	83.00
	CyMb	235	212	70.67
	MbCO	251	172	57.33
	Cy	240	134	44.67
	CO	246	118	39.33
	Mb	251	64	21.33
	Untreated	267	21	7
	BE	251	31	10.3

Ifakara blend (Ib)	CyIbCO	255	245	81.67
	CyIb	251	155	51.67
	IbCO	237	122	40.67
	Cy	236	155	51.67
	CO	260	126	42.00
	Ib	242	68	22.67
	Untreated	255	16	5.33
	BE	216	31	10.33

The columns of the table indicate the combination of treatments, total numbers of mosquitoes exposed to baited POHD, and total numbers and average numbers of dead *An. arabiensis* after exposure to the baited POHD. The treatments are abbreviated as follows: untreated: attractants without bendiocarb-treated netting, BE: bendiocarb-treated netting without attractants, CO: carbon dioxide (CO_2_), Mb: Mbita blend, Cy: cyclopentanone granules, MbCO: Mbita blend combined with CO_2_, CyMb: Mbita blend combined with cyclopentanone granules, CyMbCO: Mbita blend combined with cyclopentanone granules and CO_2_, IbCO: Ifakara blend combined with CO_2_, CyIb: Ifakara blend combined with cyclopentanone granules, CyIbCO: Ifakara blend combined with cyclopentanone granules and CO_2_.

**Table 2:** Potentiating/enhancing effects of cyclopentanone and carbon dioxide on attractiveness of synthetic blends baited POHD against *An. arabiensis*.

Blend types	Combination of treatments of POHD	Average number of dead mosquitoes	Individual treatments of POHD	Average number of dead mosquitoes	*P* values
Mbita blend (Mb)	CyMb	70.67	Mb	21.33	*P* < 0.001
			Cy	44.67	*P* < 0.001
	MbCO	57.33	Mb	21.33	*P* < 0.001
			CO	39.33	*P* < 0.001

Ifakara blend (Ib)	CyIb	51.67	Ib	22.67	*P* < 0.001
			Cy	49.33	*P*=1
	IbCO	40.67	Ib	22.67	*P* < 0.001
			CO	39.00	*P*=0.84
				

The potentiating effect is indicated by the average number of dead mosquitoes in a column of combined effects of synthetic blend and either cyclopentanone or carbon dioxide that is greater than the average number of dead mosquitoes in column of individual effects of synthetic blend. The treatments are abbreviated as follows: CyMb: Mbita blend combined with cyclopentanone, Cy: cyclopentanone alone, CO_2_: carbon dioxide alone, Mb: Mbita blend alone, Ib: Ifakara blend alone, MbCO: Mbita blend combined with CO_2_, IbCO: Ifakara blend combined with CO_2_, CyIb: Ifakara blend combined with cyclopentanone.

## Data Availability

The data used to support the findings of this study are available from the corresponding author upon request.
